# Dark blood Late Gadolinium Enhancement improves conspicuity of ablation lesions

**DOI:** 10.1186/1532-429X-18-S1-P211

**Published:** 2016-01-27

**Authors:** Peter Kellman, Laura Olivieri, Elena Grant, Charles I Berul, Kendall O'Brien, Kanishka Ratnayaka, Michael S Hansen

**Affiliations:** 1 Children's National Health System, Washington, DC USA; 2 National Heart, Lung and Blood Institute, National Institutes of Health, Bethesda, MD USA

## Background

Cardiac MR has begun to be utilized for imaging ablation lesions. Peri-procedural imaging could potentially be used to guide therapy, but this application has unique requirements and limitations. In peri-procedural imaging of acute lesions, subjects are frequently sedated and imaging should be conducted free-breathing. Furthermore, Late Gadolinium Enhancement (LGE) imaging of sub-endocardial lesions has potentially poor contrast with the adjacent bright blood pool. A dark-blood (DB) LGE sequence has previously been proposed [1], but requires a lengthy pre-scan for timing calibration and necessitates breath-holding. We have developed a free-breathing, DB LGE approach for improved blood pool contrast. DB LGE images are acquired using an IR-T2 preparation [1]. We extend this method to be used with single shot PSIR LGE and incorporate respiratory motion corrected averaging to improve image quality. Furthermore, we introduce a Bloch simulation approach to determining sequence delay times, eliminating the lengthy calibration scan. The proposed method was evaluated as part of a research study of peri-procedural cardiac MR imaging in children undergoing catheter ablation for VT.

## Methods

Free-breathing LGE imaging was performed using a single shot SSFP sequence with respiratory motion correction averaging of repeated measurements. DB LGE was implemented by adding a T2 prep between the IR preparation and the readout, which shifts the relative null times making it possible to choose delays that simultaneously null both myocardium and blood. Measured T1 values, from a rapid T1 scout, were used in a Bloch simulation of the sequence, to derive delays used for DB LGE imaging. PSIR image reconstruction was used to improve contrast and reduce dependence on inversion time (TI). By using PSIR reconstruction to preserve signal polarity, it is possible to make the blood signal negative resulting in the blood appearing darker than the myocardium. Typical imaging protocol was: SSFP readout, 50° FA, 360 × 270 × 8 mm^3^, 256 × x144 matrix, parallel imaging R=2, 8 repeated measurements, T2 prep TE 30 ms. T1-mapping was based on an 11 HB motion corrected MOLLI. Imaging was performed at 1.5T (Magnetom AERA, Siemens). The study was IRB-approved and 2 subjects consented for research. LGE images were acquired approx. 15 min following administration of Gd.

## Results

**Figure 1:**
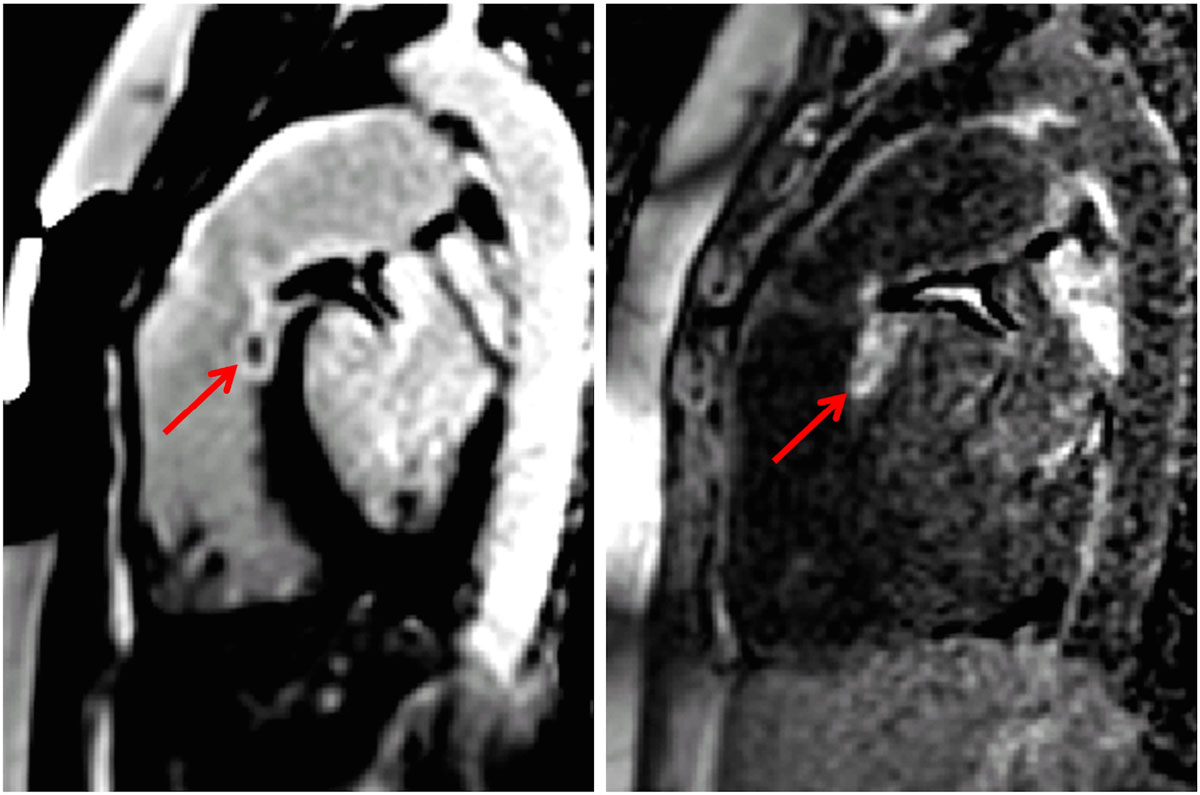
**Free-breathing PSIR LGE acquired immediately following ablation in subject 1 using bright blood (left) and dark blood (right) protocols shows RVOT ablation lesion**. Contrast between ablation lesion and adjacent blood pool increased more than 6:1.

**Figure 2:**
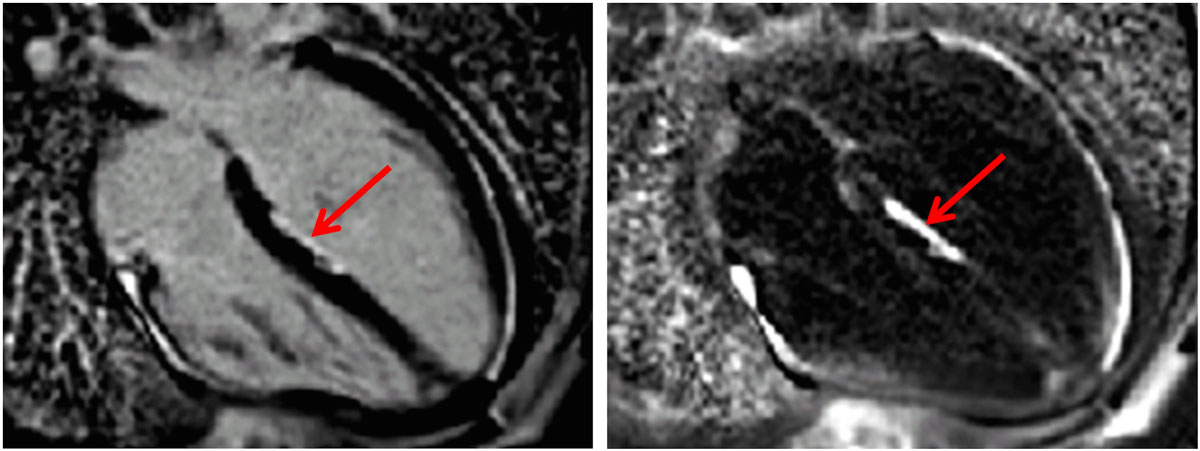
**Free-breathing PSIR LGE for subject 2 in 20-week follow-up study**. Bright blood (left) and dark blood (right) with ablation lesion in LV septum.

Subject 1 (Figure 1) was scanned immediately following ablation. Enhancement of RVOT ablation region is visible with both bright and DB protocols, but is more readily visualized with DB. Subject 2 (Figure 2) was scanned for a follow-up study at 20 wks post ablation.

## Conclusions

A robust free-breathing imaging protocol using DB PSIR LGE has been demonstrated to improve the visualization of scar from catheter ablation. This new technical development was demonstrated as an adjunct to a pilot study assessing peri-procedural imaging of anaesthetized children undergoing catheter ablation of ventricular arrhythmia.
